# Patterns of philopatry and longevity contribute to the evolution of post-reproductive lifespan in mammals

**DOI:** 10.1098/rsbl.2015.0992

**Published:** 2016-02

**Authors:** H. J. Nichols, L. Zecherle, K. Arbuckle

**Affiliations:** 1 School of Natural Science and Psychology, Liverpool John Moores University, Liverpool L3 3AF, UK; 2 Institute of Integrative Biology, University of Liverpool, Liverpool L69 7ZB, UK

**Keywords:** menopause, life-history evolution, kin-selection, dispersal, primate, cetacean

## Abstract

While menopause has long been known as a characteristic trait of human reproduction, evidence for post-reproductive lifespan (PRLS) has recently been found in other mammals. Adaptive and non-adaptive hypotheses have been proposed to explain the evolution of PRLS, but formal tests of these are rare. We use a phylogenetic approach to evaluate hypotheses for the evolution of PRLS among mammals. In contrast to theoretical models predicting that PRLS may be promoted by male philopatry (which increases relatedness between a female and her group in old age), we find little evidence that male philopatry led to the evolution of a post-reproductive period. However, the proportion of life spent post-reproductive was related to lifespan and patterns of philopatry, suggesting that the duration of PRLS may be impacted by both non-adaptive and adaptive processes. Finally, the proportion of females experiencing PRLS was higher in species with male philopaty and larger groups, in accordance with adaptive models of PRLS. We suggest that the origin of PRLS primarily follows the non-adaptive ‘mismatch’ scenario, but that patterns of philopatry may subsequently confer adaptive benefits of late-life helping.

## 1. Introduction

Menopause has long been known as a characteristic of human reproduction [1] but the existence of post-reproductive lifespan (PRLS) in other mammalian species has been recognized only relatively recently [2,3]. Post-reproductive periods of 20+ years (similar to that observed in humans) have been found in two long-lived cetacean species [4]. Shorter periods of PRLS have been identified in a wide range of mammalian species, including cetaceans, primates and rodents [2]. However, the existence of PRLS is an evolutionary paradox: natural selection would be expected to disfavour the premature cessation of reproduction. Why then is PRLS so widespread?

PRLS may have an adaptive value. For example, menopause could be favoured if mothers provide help to adult offspring, thereby increasing the production of grand-offspring [5]. Lahdenperä *et al*. [6] showed that human grandmothers are able to boost the reproductive success of their children. Similarly, in killer whales, post-reproductive females appear to extend the longevity of their adult offspring [7]. This adaptive ‘grandmother hypothesis’ for the evolution of PRLS depends on the presence of kin towards whom help can be directed.

Johnstone & Cant [8] developed a model to explain the involvement of kin selection in the evolution of prolonged PRLS in cetaceans and humans that suggested parallel routes for the evolution of PRLS. While early humans are believed to have been male-philopatric, in killer whales and short-finned pilot whales neither sex disperses and mating occurs outside the family group. These dispersal strategies are predicted to lead to an increase in relatedness to other group members throughout the lifetime of a female. In both situations, a female begins her reproductive life away from her father and other paternal relatives (either because she has dispersed or because she was the product of an extra-group mating). However, her sons remain within the social group, and hence relatedness between the female and local males (and therefore average relatedness to group members) increases over the female's lifespan, thereby leading to the evolution of an adaptive period of post-reproductive helping behaviour [8].

While the literature focuses on PRLS as an adaptive trait, it could simply be a non-adaptive by-product of other life-history traits. PRLS is of short duration in most mammals, leading to the proposal that PRLS is a consequence of mismatch in somatic versus reproductive senescence [2]. This mismatch may be more likely to occur in long-lived species as the associated variability in maximum lifespan leads to an increased probability that some individuals exceed the age by which oocytes are depleted.

Attempts to test the predictions of adaptive versus non-adaptive hypotheses for the evolution of PRLS are lacking, despite a theoretical framework for both classes of explanation [2,8]. In this study, we use a comparative approach to investigate whether natural history traits can predict the existence and extent of PRLS among mammals. If PRLS has arisen adaptively owing to kin selection, we expect sex-specific dispersal dynamics to be important in the evolution of PRLS [8]. Alternatively, if PRLS arises primarily owing to a mismatch between somatic and reproductive ageing, then we would expect PRLS to be seen in longer-lived species.

## 2. Material and methods

### (a) Data collection

A literature search was conducted to identify all mammalian species for which reliable PRLS data are available (see the electronic supplementary material for our strategy for categorizing PRLS, including caveats, and electronic supplementary material, table S1, for the data obtained). We recorded the presence or absence of PRLS, the duration of PRLS and the frequency with which PRLS is experienced in the population. For species for which we had data on the presence or absence of PRLS, we continued our literature search to obtain data on natural history variables likely to influence local relatedness (male philopatry, female philopatry and group size), and lifespan (in years), which could influence the mismatch between somatic and reproductive ageing. Only data from wild populations were used since captivity can alter the incidence and details of PRLS [9] and therefore arguably adds no information to evolutionary studies of the trait. A dated phylogenetic tree of mammals was obtained from the literature [10] and pruned in Mesquite [11] to leave the 26 mammal species for which we had PRLS data (note that we included three populations of humans in some analyses so some sample sizes were greater than 26). This pruned tree was used for all comparative analyses conducted.

### (b) Statistical analysis

#### (i) What influences the presence of post-reproductive lifespan?

We fit generalized linear mixed models (GLMMs) with a binomial error structure using Markov Chain Monte Carlo (MCMC) with an inverse gamma hyperprior to investigate whether each natural history variable (male philopatry, female philopatry, group size and lifespan) was a predictor of the presence of PRLS. We coded the absence or presence of PRLS as having states 0 and 1, respectively, and used this as our response variable. The phylogeny was included as a random effect to account for evolutionary history and these models were run in the MCMCglmm package in R [12]. To avoid over-parametrization, each model contained only one explanatory variable. Each MCMC GLMM was run for 15 million generations, the first 500 000 of which were conservatively discarded as burnin. The chain was sampled every 10 000 generations, giving 1450 posterior samples for each model.

For significant predictors of the presence/absence of PRLS, we also reconstructed ancestral states to further assess how the traits evolved with respect to each other. Ancestral state reconstruction was conducted using Bayesian stochastic mapping in Phytools [13] and inference made based on 10 000 simulations.

#### (ii) What influences the duration of post-reproductive lifespan?

We measured relative duration of PRLS as the proportion of maximum lifespan spent post-reproductive (electronic supplementary material, table S1). We tested for effects of each natural history variable (male philopatry, female philopatry, group size and lifespan) individually on the relative duration of PRLS using generalized estimating equations (GEEs), which were fitted in the ape package in R [14]. The variance–covariance matrix for the GEEs was specified based on the phylogeny, which controls for phylogenetic relationships between species by including this information within the model.

#### (iii) What influences the frequency of post-reproductive lifespan?

To investigate which factors influence the proportion of individuals that experience PRLS, we modelled this variable as a function of each natural history variable (male philopatry, female philopatry, group size and lifespan). We used GEEs to control for any influence of phylogeny, which were fit as described in the preceding section.

## 3. Results and discussion

**Figure 1.**
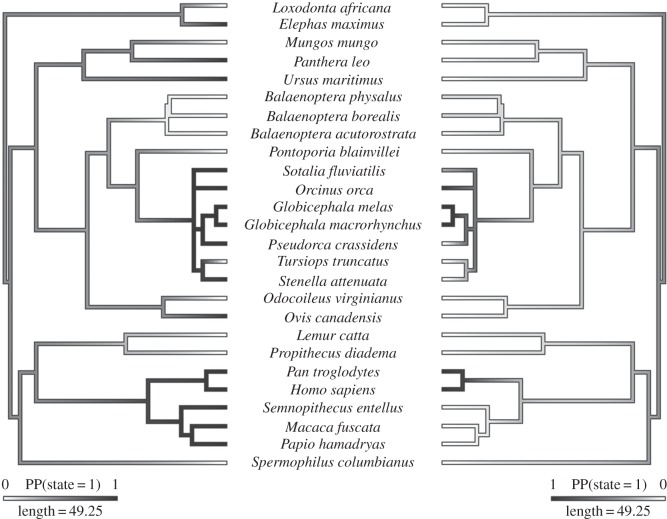
Summary of ancestral state reconstructions for PRLS (left) and male philopatry (right). Posterior probabilities (PP) of state 1 (trait is present) are represented by a greyscale gradient.

We took a phylogenetic approach to investigate natural history factors influencing PRLS in mammals with the aim of assessing whether adaptive or non-adaptive scenarios best explain its evolution. In accordance with theoretical work by Johnstone & Cant [8], we found a significant association between the presence of PRLS and male philopatry (MCMC GLMM: *β* = 340.52, *p* = 0.018, electronic supplementary material, table S2). However, Johnstone and Cant's model predicts that male philopatry is a key (but not the only) evolutionary driver of PRLS, which was not supported by our results. All five species with confirmed male philopatry exhibited PRLS, but ancestral state reconstructions suggest that PRLS evolves first, followed by male philopatry (at least in primates) (figure 1). Furthermore, 50% of the 18 species with dispersing males also exhibited PRLS, again suggesting that male philopatry is unlikely to explain the origin of PRLS in mammals.

**Figure 2.**
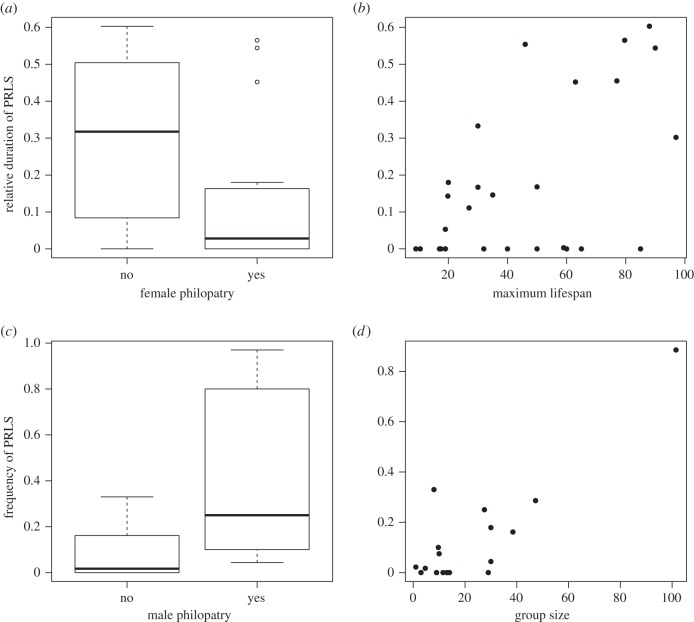
Relationships between the duration of PRLS and (*a*) female philopatry, (*b*) maximum lifespan, and between the frequency of PRLS in females and (*c*) male philopatry and (*d*) group size.

If PRLS is typically of short duration, then it is possible that patterns of philopatry are important in the evolution of an extended period of PRLS owing to their influence on kinship [4]. Supporting this, we found that species with female philopatry had significantly shorter periods of PRLS (GEE: *β* ± s.e. = −1.573 ± 0.681, *t*_1,20_ = −2.308, *p* = 0.048; electronic supplementary material, table S3; figure 2*a*), and species with male philopatry had a (non-significant) trend for increased periods of PRLS (GEE: *β* ± s.e. = 1.394 ± 0.676, *t*_1,20_ = 2.063, *p* = 0.071; electronic supplementary material, table S3). However, we note that we cannot rule out the possibility that male philopatry is associated with PRLS via its positive relationship with lifespan (pGLS (phylogenetic generalised least squares): *t* = 4.06, d.f. = 1,20, *p* = 0.001). Furthermore, factors other than dispersal patterns are expected to influence the adaptive evolution of PRLS, such as the opportunity for late-life helping and competition (which we did not fully investigate here), but Johnstone & Cant [8] propose dispersal as an important driver.

We found that the relative duration of PRLS was greater in longer-lived species (GEE: *β* ± s.e. = 0.038 ± 0.011, *t*_1,23_ = 3.482, *p* = 0.007; electronic supplementary material, table S3; figure 2*b*). While a previous study [15] found a relationship between time spent post-reproductive and lifespan, they modelled the total duration of PRLS, rather than the relative duration (as we have calculated here). Our measure is unlikely to be inherently associated with lifespan, and therefore suggests that the relationship between PRLS and lifespan is not simply an artefact of longer-living species spending more months/years post-reproductive. Instead, our results are consistent with the idea that extended PRLS can occur owing to mismatching of somatic and reproductive ageing [2]. It could also be related to selection on increased male lifespan, which could in turn lead to extended female lifespan via intersexual genetic correlations, even if they are not reproductive [2,16,17]. Disentangling these alternative non-adaptive scenarios would require detailed investigation into the evolutionary genetic constraints on lifespan across a wide range of mammals.

The proportion of females experiencing PRLS was higher in male-philopatric species (GEE: *β* ± s.e. = 1.900 ± 0.786, *t*_1,15_ = 2.418, *p* = 0.047; electronic supplementary material, table S3; figure 2*c*), suggesting that male philopatry could drive the evolution of widespread PRLS in a species [7]. We also found that PRLS was more prevalent in larger groups (GEE: *β* ± s.e. = 0.051 ± 0.014, *t*_1,15_ = 3.762, *p* = 0.0075; electronic supplementary material, table S3; figure 2*d*), possibly because these contain more philopatric young, and hence greater opportunities for helping. However, there was significant covariation between male philopatry and group size (GEE: *β* ± s.e. = 43.680 ± 18.437, *t*_1,20_ = 2.369, *p* = 0.045), making it difficult to distinguish between their effects, especially with the limited number of species for which data are currently available. Future models of the evolution of PRLS may benefit from exploring these relationships further by investigating the impacts of both group size and philopatry on kinship dynamics.

## 4. Conclusion

We tested the predictions of the most common adaptive model for the evolution of PRLS [8] and our results provide mixed support for such a model. We suggest that adaptive models such as that by Johnstone & Cant [8] may be important in explaining prolonged periods of PRLS (as they were intended to do), but do not provide a good explanation for the occurrence of shorter periods of PRLS that seem to be prevalent across mammals [2,5]. Rather, the evolutionary origin of PRLS appears primarily to follow a non-adaptive scenario such as the ‘mismatch’ hypothesis [2,18]. Patterns of philopatry may subsequently confer adaptive benefits of late-life helping, which extends the duration and frequency of PRLS [5,7,8]. Under this scenario, we suggest that the prolonged periods of PRLS found in a few species such as humans and cetaceans are a consequence of non-adaptive origins followed by adaptive evolutionary ‘tinkering’. Our results also demonstrate that for some analyses, it may be important to consider different components of PRLS separately, rather than combined in a single measure such as PrR [15]. Different factors are likely to govern the evolution of the presence, duration and frequency of PRLS and conflation of these elements in a single index limits our ability to evaluate many ideas.

## Supplementary Material

Supplementary material

## Data accessibility

Data are provided in the electronic supplementary material, table S1.
