# Supplementary material for: Patterns of philopatry and longevity contribute to the evolution of post-reproductive lifespan in mammals
Source: Biol Lett. 2016 Feb;12(2):20150992. doi: 10.1098/rsbl.2015.0992 (PMC4780556; doi:10.1098/rsbl.2015.0992)
Supplement: Supplementary material [file rsbl20150992supp1.docx]

**Supplementary Material**

***Measuring post-reproductive lifespan***

An extensive literature search was conducted to identify all wild mammalian species for which reliable PRLS data is available using the following search term in Google Scholar (where … is substituted for each mammalian taxonomic order): "Post reproductive lifespan" OR PRLS OR Menopause OR "Reproductive cessation" AND "…" . When a hit was found for a given order, the search term was repeated for each genus within that order, and resulting hits were examined individually to assess the information contained in each.

We made an effort to standardise definitions of PRLS since most of our source publications used different methods and criteria. PRLS was quantified in two ways: 1. The average interval between last birth and death (only for females whose span between last live birth and death exceeded that of their own average inter-birth interval, plus two standard deviations). 2. The maximum lifespan minus the average age at reproductive cessation (where reproductive cessation was confirmed through the cessation of menstrual cycle, changes in ovarian anatomy, low/erratic progesterone levels or the absence of pregnancies in a population). Table S1 details the ways in which PRLS was determined for each study population. We note that definition 2, which considers the maximum lifespan and average age at reproductive cessation, could feasibly lead to a bias in which better sampled populations are more likely to find a particularly long-lived individual which leads to inference of PRLS being present. However, our data suggest that this is not a problem here as the median sample size for species found to have PRLS was actually slightly lower than that for species lacking PRLS (medians of 184 and 257 respectively). Also, the overall distributions of sample size in these two groups were greatly overlapping, again suggesting that we are not seeing such a bias. Nevertheless we stress that this measure more accurately reflects the presence of PRLS in some individuals within the population, rather than implying that it is commonly experienced by individuals (the prevalence within the population was also recorded – see below).

In order to measure the duration of PRLS, we calculated the proportion of maximum lifespan spent post-reproductive. This allowed us to include all species that show PRLS. In contrast, using the mean period post-reproductive would under-estimate the occurrence of PRLS in species with high early-life mortality, even when a substantial number of females showed PRLS (Cohen 2004). We therefore chose to measure intrinsic PRLS, even if it is realised in a relatively small (but non-zero) proportion of individuals. Data were also collected from the literature on the proportion of females experiencing PRLS.

While we considered using a combined index for PRLS, such as Levitis and Lackey’s (2011) measure: PrR, this measure was not used for two reasons. First, the calculation of PrR requires life-history tables, which are not available for the vast majority of wild species. Second, by independently analysing three separate aspects of PRLS (presence, relative duration, and frequency) we are able to reveal factors that influence these components separately. In contrast, combining these different (and independent) aspects into one index, such as PrR, could easily obscure variation in one element of PRLS and also fails to acknowledge that different reasons could be behind these different aspects of the trait.

**References**

Cohen, A. A. (2004). Female post‐reproductive lifespan: a general mammalian trait. *Biological Reviews*, *79*(4), 733-750.

Levitis, D.A. and Lackey, L.B. (2011). A measure for describing and comparing postreproductive life span as a population trait. *Methods in Ecology and Evolution*, 2(5), 446-453.

**Table S1.** Life-history data obtained from the literature. Numbers after values indicate the literature source of the data. Mating system data are abbreviated to pg: polygynous, pga: polygynandrous, mg: monogamous. PRLS definitions are coded as follows: (1) average interval between last birth and death, only for females whose span between last live birth and death exceeded that of their own average inter birth interval plus 2 SD; (2) maximum lifespan minus age at reproductive cessation. For definition 2, reproductive cessation was defined through (a) cessation of menstrual/oestrous cycle (b) changes in ovarian/uterine anatomy (c) last birth/ no more pregnant females / no more females with offspring recorded (d) low/erratic progesterone levels (e) no decrease in pregnancy rate with age (f) no changes in ovarian anatomy/ oestrous cycle recorded (g) substantial decrease in pregnancy rate with age (but no data available on individual females - there may be PRLS, or alternatively, just reduced success shortly before death)

| **Species** | **PRLS present** | | **Relative duration PRLS (% max. lifespan)** | **Maximum lifespan** | **Mean group size** | **Frequency of PRLS (%)** | **Philopatry** | **Type of study population** | **Sample size** | **Definition of PRLS** | **Comments** |
| --- | --- | --- | --- | --- | --- | --- | --- | --- | --- | --- | --- |
| **Primates** | | | | | | | | | | | |
| Common marmoset  *Callithrix jacchus* | yes₁ | | 21.15_1_ | 10₁ | 9₂ | 36.4% (of females reaching middle age)_1_ | none₃, ₄ | captive | 14₁ | 1 | Not included in analyses as data is from a captive population. |
| Vervet monkey  *Chlorocebus aethiops* | no₁ | | NA | 17₁ | 40.5₂ | NA_1_ | female₂ | captive | 12₁ | 1 | Not included in analyses as data is from a captive population. |
| Western lowland gorilla  *Gorilla gorilla* | yes₅ | | 20₅ | 50₅ | 12₂ | NA | none₂ | captive | NA | 2d | Not included in analyses as data is from a captive population. |
|  | yes_1_ | | 15.11₁ | 30_1_ | 12₂ | 40% (of females reaching middle age)_1_ | none₂ | captive | 12₁ | 1 | Not included in analyses as data is from a captive population. |
|  | yes₆₃ | | 16.15₆₃ | 52₆₃ | 12₂ | 23% (of geriatric females)₆₃ | none₂ | captive | 22₆₃ | 2a | Mean age of acyclic females (43.6) used as onset PRLS. Not included in analyses as data is from a captive population. |
| Golden lion tamarin  *Leontopithecus rosalia* | yes₁ | | 32.22₁ | 12₁ | 9₂ | 47.4% (of females reaching middle age)_1_ | none₂ | captive | 21₁ | 1 | Not included in analyses as data is from a captive population. |
| Japanese macaque  *Macaca fuscata* | yes₆ | | 13.64 (mean PRLS 4.5 years)₆ | 33₆ | 47.25₆₆ | 50% (of old females)₆ | female₂ | wild (provisioned) | 33 (total females), 14 old aged females (20+ years) | 1* | * PRLS is defined as interval between last birth and death minus average period of offspring dependency (1.5 years).  Not included in analyses as data is from a provisioned population and we have data on a non-provisioned wild population (see next row). |
|  | yes**₆** | | 18 (mean PRLS 3.6 years)₆ | 20₆ | 47.25₆₆ | 28.6% (20 of 70 females experienced post reproductive lifespan)₆ | female₂ | wild (non-provisioned) | 9 old aged females (15+ years) | 1* | * PRLS is defined as interval between last birth and death minus average period of offspring dependency (1.5 years). |
| Rhesus macaque  *Macaca mulatta* | yes₁ | | 12.90_1_ | 20₁ | 30₂ | 13.2% (of females that reached middle age)_1_ | female₂ | captive | 38₁ | 1 | Not included in analyses as data is from a captive population. |
| Pigtail macaque  *Macaca nemestrina* | yes₁ | | 20.11_1_ | 20₁ | 27.5₂ | 25.6% (of females that reached middle age)_1_ | female₂ | captive | 209₁ | 1 | Not included in analyses as data is from a captive population. |
| Bonnet macaque  *Macaca radiata* | yes₁ | | 35.28_1_ | 19₁ | 27.5₂ | 3.8% (of females that reached middle age)_1_ | none₂ | captive | 26₁ | 1 | Not included in analyses as data is from a captive population. |
| Barbary macaque  *Macaca sylvanus* | yes₇ | | 21.43₇ | 28₇ | 35.5₂ | NA | female₂ | captive | NA | 2a | Not included in analyses as data is from a captive population. |
| Ring-tailed lemur  *Lemur catta* | no₆₇ | | NA | 17₆₅ | 11.5₆₄ | NA | female₇₀ | wild | 77₆₅ | 2e | No significant decline in birth rate between middle aged females (4-11 years): 80.2% and old aged females (12-17 years): 72% |
| Mouse lemur  *Microcebus murinus* | no₈ | | NA | 14₈ | 1₂ | NA | none₁₀ | captive | NA | NA | Not included in analyses as data is from a captive population. |
| Chimpanzee  *Pan troglodytes* | yes₁₁ | | 16.75 (based on average PRLS of 8.38)₁₁ | 50₁₁ | 74₁₁ | 23.5% (of old females)₁₁ | male₂ | wild | 34 old females₁₁ | 1* | *Interval between last birth and death minus period of offspring dependency (5 years)  Not included in analyses of frequency of PRLS as we couldn’t determine the proportion of the population, only “of old females”. |
|  | yes_1_ | | 19.28_1_ | 48_1_ | 74₁₁ | 60% (of females that reached middle age)_1_ | male₂ | captive | 15₁ | 1 | Not included in analyses as data is from a captive population. |
|  | no$₆₂$ | | NA | NA (last birth with 55)₆₂ | 74₁₁ | NA | male₂ | wild | 165₆₂ | NA | 47% of females that lived beyond 40 years reproduced successfully. Chimpanzee fertility declines are consistent with declines in survivorship, and healthy females maintain high birth rates late into life. Not used for analysis due to the definition used being a measure of the population, not individuals, but PRLS is an individual trait. In essence, the individual-level data should better reflect the occurrence of PRLS than population-level measures. |
| Olive baboon  *Papio anubis* | yes₁₂ | | 11.11 (fertility ceases at 24 years)₁₂ | 27₁₂ | 50₂ | NA | female₂ | wild | NA | 2a |  |
| Orangutan  *Pongo*  *pygmaeus* | yes₁ | | 18.64_1_ | 38₁ | 2₂ | 31.9% (of females that reached middle age)_1_ | none₂ | captive | 53₁ | 1 | Not included in analyses as data is from a captive population. |
| Milne-Edward’s sifaka  *Propithecus diadema edwardsi* | no₁₃ | | NA | 32₁₃ | 6₂ | NA | female₂ | wild | NA | 2f |  |
| Saddleback tamarin  *Saguinus fuscicollis* | yes₁ | | 33.54_1_ | 12₁ | 6.5₂ | 47.4% (of females that reached middle age)_1_ | none₁₄ | captive | 6₁ | 1 | Not included in analyses as data is from a captive population. |
|  | yes₁₅ | | 16.67₁₅ | 20.4₁₅ | 6.5₂ | 100% (of old females *) | none₁₄ | captive | 6₁₅ | 2a,2b,2d | *Both of 2 old females had PRLS. Not included in analyses as data is from a captive population. |
| Cotton-top tamarin  *Saguinus oedipus* | yes₁₅ | | 6.59₁₅ | 18.2₁₅ | 8₂ | 100% (of old females *) | none₁₆ | captive | 6₁₅ | 2a,2b,2d | *All 4 old females had PRLS. Not included in analyses as data is from a captive population. |
| Hanuman Langur  *Semnopithecus entellus* | Yes₁₇ | | 14.57 (5.1 average PRLS)₁₇ | 35₁₇ | 38.5₁₇ | 16.13 % (includes all observed females, not only aged females) | female₁₇ | Wild ( 1/3 of foraged food provisioned) | 31 | 1 | Relative duration PRLS calculated using average PRLS (5.1 years) |
| Squirrel monkey  *Saimiri sciureus* | yes₁ | | 17.29_1_ | 19₁ | 32₂ | 32.1% (of females that reached middle age) | female₂ | captive | 28₁ | 1 | Not included in analyses as data is from a captive population. |
| Humans  *Homo sapiens*  Ache people, Paraguay | yes₅₈ | | 45.45₅₈ | 77₅₈ | 168₅₈ | - | male₁₀₂ | wild | 292₅₈ | 2a |  |
| !Kung Bushmen, Botswana | yes₅₉ | | 60.23₅₉ | 88₅₉ | 35₆₁ | 80%₅₉ | male₁₀₂ | wild | 500₅₉ | 2a |  |
| (Krummhorn, Germany, 18^th^ & 19^th^ Century) | yes₁ | | 30.18_1_ | 97_1_ | NA | 97% (of females that reached middle age)_1_ | male₁₀₂ | wild | 106₁ | 1 |  |
| **Cetaceans** | | | | | | | | | | | |
| Antarctic minke whale  *Balaenoptera acutorostrata* | no₁₈ | | NA | 50₂₀ | 2₁₉ | NA | none₁₉ | wild | >12000₁₈ | 2e |  |
| Antarctic fin whale  *Balaenoptera physalus* | no₉₆ | | NA | 85₉₆ | 1.56₉₇ | NA | NA | wild | 1422₉₆ | 2e |  |
| *Sei whale*  *Balaenoptera borealis* | no₁₈ | | NA | 60₂₁ | 3₁₉ | NA | NA | wild | 1521₁₈ | 2e |  |
| Short-finned pilot whale  *Globicephala macrorhynchus* | yes₂₂ | | 45.24₂₂ | 63₂₂ | 27.5₁₉ | 25%₂₄ | both₂₃ | wild | 245₂₂ | 2b, 2c | Relative duration of PRLS calculated based on mean age at onset of PRLS (34.5 years) |
| Long-finned pilot whale  *Globicephala melas* | yes₂₄ | | 0.32₂₄ | 59₂₄ | 30₁₉ | 4.4% (of mature females)₂₄ | both₂₅, ₂₆ | wild | 1070₂₄ | 2a, 2b |  |
| Killer whale  *Orcinus orca Northern* | yes₂₈ | | 54.44 (50% post reproductive at 41 years)₂₈ | 90₂₈ | 9.7₂₈/ 26₈* | 10% of population₂₈ | both₂₃ | Wild | 63₂₈/ 41₂₈* | 2c | *Northern/ Southern population |
| Franciscana  *Pontoporia blainvillei* | no₃₀ | | NA | 19₃₀ | NA | NA | female₃₂ | Wild | 97₃₀ | 2f |  |
| False killer whale  *Pseudorca crassidens* | yes₁₈ | | NA | NA | 30₁₉ | 17.91% (of all mature females)₁₈ | female₆₈ | wild | 67 (mature females)₁₈ | 2c |  |
| Estuarine dolphin  *Sotalia guianensis* | yes₃₃ | | 16.67₃₃ | 30₃₄ | 12.4₃₅ | NA | NA | wild | 23₃₃ | 2b |  |
| Spinner dolphin  *Stenella longirostris* | yes₉₈ | | NA | 536₉₈ | 211₆₉ | 0.74% (of adult females)₁₀₁ | Variable₇₀ | wild | 536₉₈ | 2b | Not used as data are extremely uncertain as to whether PRLS exists in this species. Although reported to have PRLS this is based on data from non-aged individuals that could have been pathological aberrations of ovaries. |
| Spotted dolphin  *Stenella*  *attenuata* | yes₃₆ | | 55.43₃₆ | 46 (mean LS)₃₇ | 252.5₁₉ | NA | Uncertain₇₁ | wild | 257₃₆ | 2c |  |
| Bottlenose dolphin  *Tursiops truncatus* | no₁₈ | | NA | 40₁₈ | 13₁₉ | NA | female₃₈ | wild | 151₁₈ | 2e |  |
| **Perissodactyla** | | | | | | | | | | | |
| Domestic horse  *Equus caballus* | Yes₇₃ | | 6.67₇₃ | 45₇₃ | 4.52₇₂ | NA | none₇₄ | captive (domestic) | NA | 2c | Not included in analyses as data is from a captive population. |
| **Artiodactyla** | | | | | | | | | | | |
| Domestic cattle  *Bos primigenius Taurus* | yes₄₀ | | 25 | 20₄₀ | 10.5₄₁ | >50% (infertile at 15 years) | female₄₂ | captive | 152₄₀ | 2c | Not included in analyses as data is from a captive population. |
| White-tailed deer  *Odocoileus virginianus* | no₄₄ | | NA | 17.50₄₄ | 3₄₇ | NA | female₄₅ | wild | 284₄₄ | 2e |  |
| Bighorn sheep  *Ovis canadensis* | Yes₇₅ | | 5.26₇₅ | 19₇₅ | 10₇₅ | 0.75%₇₅ | female₇₈ | wild | 265₇₅ | 2c | Evidence of reproductive senescence, evidence of PRLS was indirect but retained as many individuals appeared to stop reproducing. At age 12, ~30% of females produced offspring. |
| Soay sheep  *Ovis aries* | Maybe₉₁ | | NA | 12₉₁ | NA | NA | NA | wild | 894₉₁ | 2f | Not used in analysis as data were insufficient to determine whether PRLS occurs in this species. The published data only suggest than decline in reproduction is related to within-individual changes but not whether the change is a cessation of reproduction or merely a reduction. |
| Red deer  *Cervus elaphus* | yes₉₂, ₉₃ | | > 9.52₉₃ | > 21 (females were culled at 21 years)₉₃ | 30₉₅ | 47.5% (of population )₉₃ | female₉₈ | captive | 40₉₃ | 2c | Approx 5% of females bred at age 20+. The rest had ceased reproducing due to ovarian failure (confirmed by dissection). Not included in analyses as data is from a captive population. |
|  | maybe₉₄ | | NA | 18 (very few females live beyond this age) | 30₉₅ | NA | female₉₈ | wild | 551₉₅ | 2f | The species shows a rapid decline in fertility past age 14, but it is not clear whether there is substantial PRLS. By age 17+, 20% of females were still reproducing. Not included in analyses as data are insufficient to differentiate PRLS in individuals from reduction in reproductive output with age across the population as a whole. |
| **Carnivora** | | | | | | | | | | | |
| Cat  *Felis catus* | Yes₈₂ | | 30₈₂ | 20₈₅ | Variable ₈₃ (solitary and group-living) | NA | none₈₃ | captive (domestic) | NA | NA | Relative duration PRLS is calculated using mean age at reproductive cessation (14 years). Not included in analyses as data is from a captive population. |
| Polar bear  *Ursus maritimus* | yes₄₈ | | 33.33₄₈ | 30₄₈ | 1₄₉ | 2.2%* | none₄₉ | wild | 402₄₈ | 2c | *9 non-reproductive females over age 18 in a population of 402_48_ |
| African lion  *Panthera leo* | yes₁₂ | | 14.27₁₂ | 19.83₁₂ | 4.64₇₇ | 1.7% (pers. com. Prof C Packer) | female/none₇₆ | wild | 123₁₂ | 2c |  |
| Banded Mongoose  *Mungos mungo* | no₆₁ | | NA | 10.50₆₁ | 14₆₁ | NA | none₆₁ | wild | NA | 1 |  |
| Meerkat  *Suricata suricatta* | maybe₈₀ | | NA | 12₈₀ | 16.7₈₁ | NA | none₈₁ | Wild | 42 (dominant females) ₈₀ | 2f | Substantial reproductive senescence occurs. However, females were still producing on average ~0.5 litters per year at age 12 (maximum age). Not included in analyses as data are insufficient to differentiate PRLS in individuals from reduction in reproductive output with age across the population as a whole |
| Dog  *Canis familiaris* | yes₇₃ | | 43.75₇₃ | 16 | 4₇₈ | <50%₇₉ | none₇₈ | captive (domestic)₇₃ | NA | 2c | Not included in analyses as data is from a captive population |
| **Proboscidae** | | | | | | | | | | | |
| African elephant  *Loxodonta africana* | no₅₀ | | NA | 65₅₀ | 9₅₁ | Of the 38 females in the study with well-kn own  histories who reached 50 years old, most continued to  reproduce and only nine appeared to stop breeding  with 7 years or more passing since they last gave birth.  Of the 38 females in the study with well-kn own  histories who reached 50 years old, most continued to  reproduce and only nine appeared to stop breeding  with 7 years or more passing since they last gave birth.  NA | female₅₂ | wild | 546 (38 survived reached  >50)₅₀ | 2e |  |
| Asian elephant  Elephas maximus | Yes₈₅ | | 12.5₈₅ | 79.64₈₅ | 8₈₄ | 32.95% (457/1040) live past 40 years: age when 75% of females ceased to reproduce) ₈₅ | female₈₄ | Mixed₈₄ | 1040₈₅ | Mean lifespan – mean age at last reproduction | Authors state PRLS present in species. Calculated as interval between average age at last birth and mean lifespan. This data was subdivided into wild and captive individuals, which we describe separately in the following two rows. |
|  | No/maybe₈₅ | | 17.01(54 oldest reproducing female)  54.11 (Mean age at last reproduction 29.88) ₈₅ | 65.11₈₅ | 8₈₄ | NA | female₈₄ | captive | 471₈₅ | 1* | When PRLS was calculated using oldest age at last reproduction, interval does not exceed mean IBI + 2SD. * (Mean IBI 5.99 ± 2.99 years). Not included in analyses as data is from a captive population |
|  | Yes ₈₅ | | 56.49 (mean age at last reproduction 34.65)₈₅ | 79.64₈₅ | 8₈₄ | 32.95% ₈₅ | female₈₄ | wild | 569₈₅ | 1* | Interval between oldest age at last reproduction and death exceeds mean IBI+2SD for wild population. *Mean IBI 5.99 ± 2.99 years |
| **Lagomorpha** | | | | | | | | | | | |
| Domestic rabbit  *Oryctolagus cuniculus* | yes₈₆ | | 66.67₈₆ | 15₈₆ | 7₉₀ | NA | female₈₇ | captive (domestic) | NA | 2 (method NR) | Not included in analyses as data is from a captive population |
| **Rodontidae** | | | | | | | | | | | |
| Lab mouse  *Mus musculus* | | yes₇₃ | 60.00₇₃ | 4.17₇₃ | 1₈₈ | NA | none$₈₈$ | captive (domestic) | NA | 2c | Not included in analyses as data is from a captive population |
| Lab rat  *Rattus norvegicus* | | yes₇₆ | 52.00₇₃ | 4.17₇₃ | Variable (solitary when food dispersed, in urban environments mean groups of 22.5) ₈₉ | NA | none₉₀ | captive (domestic) | NA | 2c | Not included in analyses as data is from a captive population |
| Chinese hamster  *Cricetulus griseus* | | no₅₃ | NA | 1.75₅₃ | NA | NA | NA | captive | 25 (aged females) ₅₃ | 2c, g | Signs of reproductive senscence (reduced litter size, 23% of aged females failed to ovulate). However, 60% of aged females still reproduced no oocyte depletion in aged females. Not included in analyses as data is from a captive population |
| Columbian ground squirrel  *Spermophilus columbianus* | | no₅₄ | NA | 9₅₄ | 29₅₇ | NA | female₅₅ | wild | 229₅₄ | 2g | 60% of older females (6-9 years) weaned litters successfully |

**References for Table S1**

1. Caro, T. M., Sellen, D. W., Parish, A., Frank, R., Brown, D. M., Voland, E., Mulder, M. B. 1995 Termination of reproduction in nonhuman and human female primates. *International Journal of Primatology*, **16**, 205-220.
2. Rowe, N., Goodall, J., Mittermeier, R. 1996 *The pictorial guide to the living primates* (Vol. 9). New York: Pogonias Press.
3. Pontes, A. R. M., da Cruz, M. A. O. M. 1995 Home range, intergroup transfers, and reproductive status of common marmosets *Callithrix jacchus* in a forest fragment in North-Eastern Brazil. *Primates*, **36**, 335-347.
4. de Sousa, M. B. C., da Rocha Albuquerque, A. C. S., Yamamoto, M. E., Araújo, A., de Fátima Arruda, M. 2009 Emigration as a reproductive strategy of the common marmoset (*Callithrix jacchus*). *The Smallest Anthropoids*. Springer US.
5. Walker, M. L., & Herndon, J. G. 2008 Menopause in nonhuman primates?.*Biology of reproduction*, **79**, 398-406.
6. Pavelka, M. S. M., Fedigan, L. M., & Zohar, S. (2002). Availability and adaptive value of reproductive and postreproductive Japanese macaque mothers and grandmothers. *Animal Behaviour*, *64*(3), 407-414.
7. Paul, A., Kuester, J., Podzuweit, D. 1993 Reproductive senescence and terminal investment in female Barbary macaques (*Macaca sylvanus*) at Salem. *International Journal of Primatology*, **14**, 105-124.
8. Bons, N., Rieger, F., Prudhomme, D., Fisher, A., Krause, K. H. 2006 Microcebus murinus: a useful primate model for human cerebral aging and Alzheimer's disease?. *Genes, Brain and Behavior*, **5**, 120-130.
9. Radespiel, U. 2000 Sociality in the gray mouse lemur (*Microcebus murinus*) in northwestern Madagascar. *American Journal of Primatology*, **51**, 21-40.
10. Radespiel, U., Lutermann, H., Schmelting, B., Bruford, M. W., & Zimmermann, E. 2003 Patterns and dynamics of sex-biased dispersal in a nocturnal primate, the grey mouse lemur, *Microcebus murinus*. *Animal Behaviour*, **65**, 709-719.
11. Nishida, T., Corp, N., Hamai, M., Hasegawa, T., Hiraiwa‐Hasegawa, M., Hosaka, K., Zamma, K. 2003 Demography, female life history, and reproductive profiles among the chimpanzees of Mahale. *American Journal of Primatology*, **59**, 99-121.
12. Packer, C., Tatar, M., & Collins, A. 1998 Reproductive cessation in female mammals. *Nature*, **392**, 807-811. And personal communication, Parker,C.
13. Wright, P., King, S., Baden, A., Jernvall, J. 2008 Aging in wild female lemurs: sustained fertility with increased infant mortality. 17-28.
14. Garber, P. A. 1997 One for all and breeding for one: cooperation and competition as a tamarin reproductive strategy. *Evolutionary Anthropology: Issues, News, and Reviews*, **5**, 187-199.
15. Tardif, S. D., & Ziegler, T. E. 1992 Features of female reproductive senescence in tamarins (*Saguinus spp*.), a New World primate. *Journal of reproduction and fertility*, **94**, 411-421.
16. Savage, A., Giraldo, L. H., Soto, L. H., Snowdon, C. T. 1996 Demography, group composition, and dispersal in wild cotton‐top tamarin (*Saguinus oedipus*) groups. *American Journal of Primatology*, **38**, 85-100.
17. Sommer, V., Srivastava, A., Borries, C. 1992 Cycles, sexuality, and conception in free‐ranging langurs (*Presbytis entellus*). *American Journal of Primatology*, **28**, 1-27.
18. Marsh, H., Kasuya, T. 1986 Evidence for reproductive senescence in female cetaceans. *Report of the International Whaling Commission*, **8**, 57-74.
19. Evans, P. G. 1987 *The natural history of whales & dolphins*. Helm.
20. Carey, J., Judge, D. 2002 Longevity records: life spans of mammals, birds, amphibians, reptiles, and fish. *On-line). Max Planck Institute for Demographic Research. Accessed June*, **13**, 2005. **Cited in** Samuels, D. C. 2005 Life span is related to the free energy of mitochondrial DNA. *Mechanisms of ageing and development*, **126**, 1123-1129.
21. Kanda, N., Goto, M., Pastene, L. A. 2006 Genetic characteristics of western North Pacific sei whales, *Balaenoptera borealis*, as revealed by microsatellites. *Marine Biotechnology*, **8**, 86-93.
22. Kasuya, T., Marsh, H. 1984 Life history and reproductive biology of the short-finned pilot whale, *Globicephala macrorhynchus*, off the Pacific coast of Japan. *Report of the International Whaling Commission,* **6**, 259-310.
23. Johnstone, R. A., & Cant, M. A. 2010 The evolution of menopause in cetaceans and humans: the role of demography. *Proceedings of the Royal Society B: Biological Sciences*, **277**, 3765-3771.
24. Martin, A. R., Rothery, P. 1993 Reproductive parameters of female long-finned pilot whales (*Globicephala melas*) around the Faroe Islands. *Rep. Int. Whal. Comm.,* **14**, 263-304.
25. De Stephanis, R., Verborgh, P., Pérez, S., Esteban, R., Minvielle-Sebastia, L., Guinet, C. 2008 Long-term social structure of long-finned pilot whales (*Globicephala melas*) in the Strait of Gibraltar. *acta ethologica*, **11**, 81-94.
26. Amos, B., Bloch, D., Desportes, G., Majerus, T. M., Bancroft, D. R., Barrett, J. A., Dover, G. A. 1993 A review of the molecular evidence relating to social organisation and breeding system in the long-finned pilot whale. *Report of the International Whaling Commission,* **14**, 209-217.
27. Ward, E. J., Parsons, K., Holmes, E. E., Balcomb III, K. C., Ford, J. K., Altenburger, A., Gunz, P. 2009 The role of menopause and reproductive senescence in a long-lived social mammal. *Frontiers in zoology*, **6**.
28. Olesiuk, P. F., Ellis, G. M., & Ford, J. K. 2005 *Life history and population dynamics of northern resident killer whales (Orcinus orca) in British Columbia*. Canadian Science Advisory Secretariat.
29. Pilot, M., Dahlheim, M. E., Hoelzel, A. R. 2010 Social cohesion among kin, gene flow without dispersal and the evolution of population genetic structure in the killer whale (*Orcinus orca*). *Journal of evolutionary biology*, **23**, 20-31.
30. Danilewicz, D. 2003 Reproduction of female franciscana (Pontoporia blainvillei) in Rio Grande do Sul, southern Brazil. *Latin American Journal of Aquatic Mammals*, ***2***, 67-78.
31. Panebianco, M. V., Negri, M. F., Cappozzo, H. L. 2012. Reproductive aspects of male franciscana dolphins (*Pontoporia blainvillei*) off Argentina. *Animal Reproduction Science*, **131**, 41-48.
32. Costa-Urrutia, P., Abud, C., Secchi, E. R., Lessa, E. P. 2012 Population genetic structure and social kin associations of Franciscana dolphin*, Pontoporia blainvillei*. *Journal of Heredity*, **103**, 92-102.
33. Weber Rosas, F. C., Monteiro-Filho, E. L. 2002 Reproduction of the estuarine dolphin (*Sotalia guianensis*) on the coast of Paraná, southern Brazil.*Journal of Mammalogy*, ***83***, 507-515.
34. Rosas, F. C. W., Barreto, A. S., Monteiro, E. L. D. 2003. Age and growth of the estuarine dolphin (*Sotalia guianensis*) (Cetacea, Delphinidae) on the Paraná coast, southern Brazil. *Fishery Bulletin*, **101**, 377-383.
35. Santos, M. D. O., Rosso, S. 2007 Ecological aspects of marine tucuxi dolphins (*Sotalia guianensis*) based on group size and composition in the Cananéia estuary, southeastern Brazil. *Latin American Journal of Aquatic Mammals*, **6**(, 71-82.
36. Kasuya, T. 1985 Effect of exploitation on reproductive parameters of the spotted and striped dolphins off the Pacific coast of Japan. *Scientific Reports of the Whales Research Institute*, **36**, 107-138. **Cited in** Marsh, H., & Kasuya, T. 1986 Evidence for reproductive senescence in female cetaceans. *Report of the International Whaling Commission*, **8**, 57-74.
37. Nowak, R. M. (Ed.). 2003 *Walker's marine mammals of the world*. JHU Press.
38. Möller, L. M., Beheregaray, L. B. 2004 Genetic evidence for sex‐biased dispersal in resident bottlenose dolphins (*Tursiops aduncus*). *Molecular Ecology*, **13**, 1607-1612.
39. Scott, M. D., Wells, R. S., Irvine, A. B. 1990 A long-term study of bottlenose dolphins on the west coast of Florida 11. *The Bottlenose Dolphin*, **235**.
40. Erickson, B. H., Reynolds, R. A., Murphree, R. L. 1976 Ovarian characteristics and reproductive performance of the aged cow. *Biology of Reproduction*, **15**, 555-560.
41. Hernández, L., Barral, H., Halffter, G., & Colón, S. S. 1999 A note on the behavior of feral cattle in the Chihuahuan Desert of Mexico. *Applied Animal Behaviour Science*, **63**, 259-267.
42. Reinhardt, V., Reinhardt, A. 1981 Cohesive relationships in a cattle herd (*Bos indicus). Behaviour*, **77**, 121-151.
43. Lazo, A. 1995 Ranging behaviour of feral cattle (*Bos taurus*) in Donana National Park, SW Spain. *Journal of Zoology*, **236**, 359-369.
44. DelGiudice, G. D., Lenarz, M. S., Powell, M. C. 2007 Age-specific fertility and fecundity in northern free-ranging white-tailed deer: evidence for reproductive senescence? *Journal of Mammalogy*, **88**, 427-435.
45. Jarman, P. 1974 The social organisation of antelope in relation to their ecology. *Behaviour*, **48**, 215-267.
46. DeYoung, R. W., Demarais, S., Gonzales, R. A., Honeycutt, R. L., Gee, K. L. 2002 Multiple paternity in white-tailed deer (*Odocoileus virginianus*) revealed by DNA microsatellites. *Journal of Mammalogy*, **83**, 884-892.
47. Lagory, K. E. 1986 Habitat, group size, and the behaviour of white-tailed deer.*Behaviour*, **98**, 168-179.
48. Ramsay, M. A., Stirling, I. 1988 Reproductive biology and ecology of female polar bears (*Ursus maritimus*). *Journal of Zoology*, **214**, 601-633.
49. Derocher, A. E., Andersen, M., Wiig, Ø., Aars, J. 2010 Sexual dimorphism and the mating ecology of polar bears (*Ursus maritimus*) at Svalbard. *Behavioral Ecology and Sociobiology*, **64**, 939-946.
50. Moss, C. J. 2001 The demography of an African elephant (*Loxodonta africana*) population in Amboseli, Kenya. *Journal of Zoology*, **255**, 145-156.
51. Leuthold, W. 1976 Age structure of elephants in Tsavo National Park, Kenya. *Journal of Applied Ecology*, 435-444.
52. Rasmussen, L. E. L., Schulte, B. A. 1998 Chemical signals in the reproduction of Asian (*Elephas maximus*) and African (*Loxodonta africana*) elephants. *Animal Reproduction Science*, **53**, 19-34.
53. Parkening, T. A. 1982 Reproductive senescence in the Chinese hamster (*Cricetulus griseus*). *Journal of Gerontology*, **37**, 283-287.
54. Broussard, D. R., Risch, T. S., Dobson, F. S., Murie, J. O. 2003 Senescence and age‐related reproduction of female Columbian ground squirrels. *Journal of Animal Ecology*, **72**, 212-219.
55. Wiggett, D. R., & Boag, D. A. 1989 Intercolony natal dispersal in the Columbian ground squirrel. *Canadian Journal of Zoology*, **67**, 42-50.
56. Raveh, S., Heg, D., Viblanc, V. A., Coltman, D. W., Gorrell, J. C., Dobson, F. S., …Neuhaus, P. 2011 Male reproductive tactics to increase paternity in the polygynandrous Columbian ground squirrel (*Urocitellus columbianus*).*Behavioral Ecology and Sociobiology*, **65**, 695-706.
57. Fairbanks, B., Dobson, F. S. 2007 Mechanisms of the group-size effect on vigilance in Columbian ground squirrels: dilution versus detection. *Animal Behaviour*, **7**, 115-123.
58. Hill, K. R., Hurtado, A. M. 1996 *Ache life history: The ecology and demography of a foraging people*. Transaction Publishers. **Cited in** Cohen, A. A. 2004 Female post‐reproductive lifespan: a general mammalian trait. *Biological Reviews*, **79**, 733-750.
59. Howell, N. 1979 Demography of the Dobe !Kung, New York: Academic Press
60. Draper, P. 1974 Crowding among hunter-gatherers: the !Kung Bushmen. *Crowding and Behavior*, 226.
61. Personal communication, Vitikainen, E., based on a 20 year study of a population of over 2000 individuals.
62. Thompson, M. E., Jones, J. H., Pusey, A. E., Brewer-Marsden, S., Goodall, J., Marsden, D., Wrangham, R. W. 2007 Aging and fertility patterns in wild chimpanzees provide insights into the evolution of menopause. Current Biology, **17**, 2150-2156.
63. Margulis, S. W., Atsalis, S., Bellem, A., Wielebnowski, N. 2007 Assessment of reproductive behavior and hormonal cycles in geriatric western Lowland gorillas. Zoo biology, **26**, 117-139.
64. Gould, L., Sussman, R. W., & Sauther, M. L. 2003 Demographic and life‐history patterns in a population of ring‐tailed lemurs (Lemur catta) at Beza Mahafaly Reserve, Madagascar: A 15‐year perspective. American Journal of Physical Anthropology, **120**, 182-194.
65. Ichino, S., Soma, T., Miyamoto, N., Chatani, K., Sato, H., Koyama, N., Takahata, Y. 2015 Lifespan and reproductive senescence in a free-ranging ring-tailed lemur (Lemur catta) population at Berenty, Madagascar. Folia Primatologica, **86**, 134-139.
66. Takasaki, H. 1981 Troop size, habitat quality, and home range area in Japanese macaques. Behavioral Ecology and Sociobiology, **9**, 277-281.
67. Gould, L. 1997 Intermale affiliative behavior in ringtailed lemurs (Lemur catta) at the Beza-Mahafaly Reserve, Madagascar. Primates, **38**, 15-30.
68. Foote, A. D. 2008 Mortality rate acceleration and post-reproductive lifespan in matrilineal whale species. Biology letters, **4**, 189-191.
69. Karczmarski, L., Würsig, B., Gailey, G., Larson, K. W., Vanderlip, C. 2005 Spinner dolphins in a remote Hawaiian atoll: social grouping and population structure. Behavioral Ecology, **16**, 675-685.
70. Andrews, K. R., Karczmarski, L., Au, W. W., Rickards, S. H., Vanderlip, C. A., Bowen, B. W., ...Toonen, R. J. 2010 Rolling stones and stable homes: social structure, habitat diversity and population genetics of the Hawaiian spinner dolphin (Stenella longirostris). Molecular Ecology, **19**, 732-748.
71. Courbis, S. S. 2011 *Population structure of island-associated pantropical spotted dolphins (Stenella attenuata) in Hawaiian waters*. Portland State University.
72. Linklater, W. L., Cameron, E. Z., Stafford, K. J., Veltman, C. J. 2000 Social and spatial structure and range use by Kaimanawa wild horses (Equus caballus: Equidae). *New Zealand Journal of Ecology*, 139-152.
73. vom Saal, F. S., Finch, C. E., Nelson, J. F. 1994 Natural history and mechanisms of reproductive aging in humans, laboratory rodents, and other selected vertebrates. *The physiology of reproduction*, **2**, 1213-1314.
74. Monard, A. M., Duncan, P. 1996 Consequences of natal dispersal in female horses. *Animal behaviour*, **52**, 565-579.
75. Berube, C. H., Festa-Bianchet, M., Jorgenson, J. T. 1999 Individual differences, longevity, and reproductive senescence in bighorn ewes. *Ecology*, **80**, 2555-2565.
76. Pusey, A. E., Packer, C. 1987 The evolution of sex-biased dispersal in lions. *Behaviour*, **101**, 275-310.
77. Mosser, A., Packer, C. 2009 Group territoriality and the benefits of sociality in the African lion, Panthera leo. *Animal Behaviour*, **78**, 359-370.
78. Boitani, L., Ciucci, P. 1995 Comparative social ecology of feral dogs and wolves. *Ethology Ecology & Evolution*, ***7***, 49-72.
79. Cohen, A. A. 2004 Female post-reproductive lifespan: a general mammalian trait. *Biological Reviews*, **79**, 733-750.
80. Sharp, S. P., Clutton‐Brock, T. H. 2010 Reproductive senescence in a cooperatively breeding mammal. *Journal of Animal Ecology*, **79**, 176-183.
81. Clutton-Brock, T. H., Hodge, S. J., Flower, T. P. 2008 Group size and the suppression of subordinate reproduction in Kalahari meerkats. *Animal Behaviour*, **76**, 689-700.
82. Marshall, F. H. A. 1964 *The Physiology of Reproduction, 3rd Edn*. Longmans, London. **Cited in** Cohen, A. A. 2004 Female post‐reproductive lifespan: a general mammalian trait. *Biological Reviews*, **79**, 733-750.
83. Jones, E., Coman, B. J. 1982 Ecology of the Feral Cat, Felis catus (L.), Souht-Eastern Australia III. Home Ranges and Population Ecology in Semiarid North-West Victoria. *Wildlife Research*, **9**, 409-420.
84. Vidya, T. N. C., Sukumar, R. 2005 Social organization of the Asian elephant (Elephas maximus) in southern India inferred from microsatellite DNA. *Journal of Ethology*, **23**, 205-210.
85. Lahdenperä, M., Mar, K. U., Lummaa, V. 2014 Reproductive cessation and post-reproductive lifespan in Asian elephants and pre-industrial humans. *Frontiers in zoology*, **11**, 1-14.
86. Comfort, A. 1979 *The Biology of Senescence*, 3rd Edn., New York: Elsevier, **Cited in** Cohen, A. A. 2004 Female post‐reproductive lifespan: a general mammalian trait. *Biological Reviews*, **79**, 733-750.
87. Surridge, A. K., Bell, D. J., Hewitt, G. M. 1999 From population structure to individual behaviour: genetic analysis of social structure in the European wild rabbit (Oryctolagus cuniculus). *Biological Journal of the Linnean Society*, **68**, 57-71.
88. Latham, N., Mason, G. 2004 From house mouse to mouse house: the behavioural biology of free-living Mus musculus and its implications in the laboratory. *Applied Animal Behaviour Science*, **86**, 261-289.
89. Barnett, S. A., Spencer, M. M. 1951 Feeding, social behaviour and interspecific competition in wild rats. *Behaviour*, 229-242.
90. Lynn, D. A., Brown, G. R. 2009 The ontogeny of exploratory behavior in male and female adolescent rats (*Rattus norvegicus*). *Developmental psychobiology*, **51**, 513.
91. Hayward, A. D., Wilson, A. J., Pilkington, J. G., Clutton‐Brock, T. H., Pemberton, J. M., Kruuk, L. E. 2013 Reproductive senescence in female Soay sheep: variation across traits and contributions of individual ageing and selective disappearance. *Functional Ecology*, **27**, 184-195.
92. Fisher M.W., McLeod B.J., Mockett BG, Moore GH, Drew K.R. 1996 Reproductive senescence in aged red deer hinds, *Proceedings of the New Zealand Society of Animal Production*, **56**, 344-346
93. Fisher, M. W., McLeod, B. J., Heath, D. A., Lun, S., Hurst, P. R. 2000 Role of ovarian failure in reproductive senescence in aged red deer (Cervus elaphus) hinds. *Journal of reproduction and fertility*, **120**, 211-216.
94. Nussey, D. H., Kruuk, L. E., Morris, A., Clements, M. N., Pemberton, J. M., Clutton‐Brock, T. H. 2009 Inter‐and intrasexual variation in aging patterns across reproductive traits in a wild red deer population. *The American Naturalist*, **174**, 342-357.
95. Coulson, T., Albon, S., Guinness, F., Pemberton, J., Clutton-Brock, T. 1997 Population substructure, local density, and calf winter survival in red deer (Cervus elaphus). *Ecology*, **78**, 852-863.
96. Mizroch, S. A. 1981 Analysis of some biological parameters of the Antarctic fin whale (Balaenoptera physalus). *Reports of the International Whaling Commision*, **31**, 425–434
97. Notarbartolo-di-Sciara, G., Zanardelli, M., Jahoda, M., Panigada, S., Airoldi, S. 2003 The fin whale Balaenoptera physalus in the Mediterranean Sea. *Mammal Review*, **33**, 105-150.
98. Perrin, W. F., Holts, D. B., Miller, R. B 1977 Growth and reproduction of the eastern spinner dolphin, a geographical form of Stenella longirostris in the eastern tropical Pacific. *Fishery bulletin* **75**, 725-750.
99. Cant, M. A., Johnstone, R. A. 2008 Reproductive conflict and the separation of reproductive generations in humans. *Proceedings of the National Academy of Sciences*, **105**, 5332-5336.

**Table S2.** Results from MCMC GLMMs testing for effects of four natural history variables on the presence/absence of PRLS. We coded the absence or presence of PRLS as having states 0 and 1 respectively and used this as our response variable. Estimated coefficients and 95% confidence intervals are given, and significant predictors are highlighted in bold. N is the number of species included in the model. Species that had missing data for a particular variable were excluded from the relevant models (i.e. those models that included that variable). We considered a variable to be a significant predictor of the presence of PRLS when P≤0.05.

| natural history variable | β | lower 95% CI | upper 95% CI | P | N |
| --- | --- | --- | --- | --- | --- |
| Maximum lifespan | 3.186 | -1.872 | 9.211 | 0.190 | 27 |
| Group size | 1.740 | -0.062 | 3.504 | 0.073 | 26 |
| **Male philopatry** | **340.523** | **39.603** | **632.792** | **0.018** | **25** |
| Female philopatry | -59.950 | -375.380 | 292.930 | 0.692 | 25 |

**Table S3.** Results from GEEs testing for effects of four natural history variables on the relative duration of PRLS and on the frequency with which PRLS is experience in the population. Estimated coefficients (± SE) are given, and significant predictors are highlighted in bold. Species that had missing data for a particular variable were excluded from the relevant models (i.e. those models that included that variable). N is the number of species included in the model.

| Response term | Natural history variable | β±SE | t | P | N |
| --- | --- | --- | --- | --- | --- |
| Relative duration of PRLS | **Maximum lifespan** | **0.038±0.011** | **3.482** | **0.007** | **25** |
|  | Group size | 0.009±0.005 | 1.841 | 0.100 | 24 |
|  | Male philopatry | 1.394±0.676 | 2.063 | 0.071 | 22 |
|  | **Female philopatry** | **-1.573±0.681** | **-2.308** | **0.048** | **22** |
| Frequency with which PRLS is experienced in population (proportion of females that experience PRLS) | Maximum lifespan | 0.0376±0.0159 | 2.364 | 0.052 | 16 |
|  | **Group size** | **0.0515±0.0137** | **3.762** | **0.007** | **17** |
|  | **Male philopatry** | **1.900±0.786** | **2.418** | **0.047** | **17** |
|  | Female philopatry | -0.914±0.828 | -1.104 | 0.307 | 17 |
